# Salt Tolerance Potential in Onion: Confirmation through Physiological and Biochemical Traits

**DOI:** 10.3390/plants11233325

**Published:** 2022-12-01

**Authors:** Satish Kumar Sanwal, Hari Kesh, Arvind Kumar, Bhanu Kumar Dubey, Anil Khar, Youssef Rouphael, Pradeep Kumar

**Affiliations:** 1 ICAR—Central Soil Salinity Research Institute, Karnal 132001, India; 2 National Horticultural Research and Development Foundation, Karnal 132001, India; 3 ICAR—Indian Agricultural Research Institute, New Delhi 110012, India; 4 Department of Agricultural Sciences, University of Naples Federico II, 80055 Portici, Italy; 5 ICAR—Central Arid Zone Research Institute, Jodhpur 342003, India

**Keywords:** salinity, oxidative stress, genotypes, accessions, antioxidants

## Abstract

Production of many crops, including onion, under salinity is lagging due to limited information on the physiological, biochemical and molecular mechanisms of salt stress tolerance in plants. Hence, the present study was conducted to identify salt-tolerant onion genotypes based on physiological and biochemical mechanisms associated with their differential responses. Thirty-six accessions were evaluated under control and salt stress conditions, and based on growth and bulb yield. Results revealed that plant height (6.07%), number of leaves per plant (3.07%), bulb diameter (11.38%), bulb yield per plant (31.24%), and total soluble solids (8.34%) were reduced significantly compared to control. Based on percent bulb yield reduction, seven varieties were classified as salt tolerant (with <20% yield reduction), seven as salt-sensitive (with >40% yield reduction) and the remaining as moderately tolerant (with 20 to 40% yield reduction). Finally, seven salt-tolerant and seven salt-sensitive accessions were selected for detailed study of their physiological and biochemical traits and their differential responses under salinity. High relative water content (RWC), membrane stability index (MSI), proline content (PRO), and better antioxidants such as super oxide dismutase (SOD), peroxidase (POX), catalase (CAT), and ascorbate peroxidase (APX) were observed in tolerant accessions, viz. POS35, NHRDF Red (L-28), GWO 1, POS36, NHRDF Red-4 (L-744), POS37, and POS38. Conversely, increased malondialdehyde (MDA) and hydrogen peroxide (H_2_O_2_) content, reduced activity of antioxidants, more membrane injury, and high Na^+^/K^+^ ratio were observed in sensitive accessions, viz. ALR, GJWO 3, Kalyanpur Red Round, NHRDF Red-3 (L-652), Agrifound White, and NHRDF (L-920). Stepwise regression analysis identified bulb diameter), plant height, APX, stomatal conductance (gS), POX, CAT, MDA, MSI, and bulb Na^+^/K^+^ ratio as predictor traits accounting for maximum variation in bulb yield under salinity. The identified seven salt-tolerant varieties can be used in future onion breeding programs for developing tolerant genotypes for salt-prone areas.

## 1. Introduction

Onion is second most important vegetable crop after tomato with global production of 104.6 million tonnes from 5.48 million hectare area [1]. Asia accounts for the maximum share (66.8%) of total onion production in the world, followed by Africa (13.5%), Europe (9.9%), the Americas (9.6), and Oceania (0.3%) [1]. Maximum bulb onion production (26.74 million tonnes) in India consists of a 1.43 million hectare area with a productivity of 18.64 t/ha, which is less than other countries, viz. Republic of Korea (79.6 t/ha), USA, Australia, Spain, and Japan. In India, onions are grown in a wide range of climatic conditions [2]. Onion is consumed both fresh as well as in the form of processed products, owing to its nutritional and medicinal properties [3]. Onion contains bioactive compounds (quercetin, rutin etc.) and its regular consumption reduces the risk of cancer, heart diseases, diabetes, and production of reactive oxygen species [4]. Most onions in India are produced during the post-monsoon season; owing to shallow roots, onions are highly vulnerable to climatic conditions such as salinity and drought because of inadequate rainfall and low moisture content [5]. Onion is a salt-sensitive crop and because of its threshold level (1.4 dS m^−1^), the per unit increase in EC reduces 18.52% of the tuber yield [6]. The world’s 950 mha area of land (10%), 300 mha cultivated land (20%), and 230 m ha (50%) irrigated land are distressed by salt stress. Annually, about a USD 12 billion loss is estimated because of salt stress, which adversely affects agriculture production. Salinity changes the morpho-physiological, biochemical, and metabolic processes and affects onion growth and development [7]. Soil salinity increases the Na^+^ and Cl^−^ concentration in different plant parts, which ultimately influences the ionic activity in plant cells [8]. Salt stress-induced oxidative stress leads to leakage of ions, plasmolysis, membrane damage, disturbed nutrient flux, and ROS detoxification systems. These alterations adversely affect respiration, photosynthesis, hormonal balance, antioxidants activity, water use efficiency, transpiration, and plasma membrane functioning [9]. The adverse effect of salt stress on onion morphology includes reduced bulb weight, bulb diameter, plant height, leaf number per plant, root growth, and crop cycle [6,10]. High salinity decreases the production of large bulbs and maximum decreases were observed at 2.8 dS m^−1^ [11]. Plants develop different strategies such as avoidance and tissue tolerance to reduce the negative effects of salt stress. Plants also produce various compatible solutes such as glycine betaine, proline, and sugars to regulate osmotic pressure [12]. Likewise, plants produce proteins and antioxidants to counter the negative effect of reactive oxygen species. Tissue tolerance protects the plant cells by executing ion compartmentalization in plant tissues [13]. A low Na^+^/K^+^ ratio also plays a significant role in balancing the osmotic pressure and membrane stability [7].

The present study was initiated because there is not much literature on the effect of salinity stress on Indian short-day onions. To develop a breeding strategy for the development of salinity stress-tolerant onions, the basic step is to evaluate the onion accessions and to identify the promising accessions for formulating future strategies. 

In view of the scant information on the effect of salinity stress on Indian short-day onions, present study was initiated with the objective to determine the effect of salinity stress on growth and development of onions. Keeping in view this objective, we formulated and tested three research hypothesis, i.e., (i) whether salinity stress negatively affects the bulb yield and yield associated traits in onion, (ii) whether salt tolerant and salt sensitive accessions differ in physicochemical responses?, and (iii) of the most important parameters (morphological, physiological, and biochemical), which ones need to be considered for the screening and development of salt tolerant onion cultivars? To achieve these objectives, thirty-six onion cultivars were considered in the present investigation.

## 2. Materials and Methods

### 2.1. Characterization and Evaluation of Onion Accessions

Thirty-six diverse Indian short-day onion accessions including the released cultivars and some of the promising advance lines were collected from the National Horticulture Research and Development Foundation (NHRDF), Karnal; ICAR-Directorate of Onion and Garlic Research, Pune; and ICAR-Indian Agricultural Research Institute (IARI), New Delhi, India (Table 1). Seeds were sown in nursery beds and seedlings were transplanted in three replications using randomized complete block design (RCBD) under control and under saline conditions (ECiw 7 dS m^−1^) in microplots during the rabi season of 2019–20.

In each replication, 20 plants were maintained following a plant geometry of 15 × 10 cm. Soil samples were collected before sowing and after harvesting of the crop of both the treatments and analysed (Table 2). For saline treatment, natural saline ground water (ECiw~18 dS m^−1^) was used for the preparation of desired saline water salinity (ECiw 7 dS m^−1^), whereas the normal water of ECiw~1.04 dS m^−1^ was used for control treatment. Seven days after transplanting, the irrigation was given as per treatment and subsequent irrigation was given as per crop need on the basis of 100% evapotranspiration (ET), and a total of 17 irrigations were applied during the whole crop cycle. After soil analysis, the required dose of NPK was applied and the recommended packages of practices were followed for growing a good crop. As per recommendations, 50% nitrogen and 100% phosphorus and potassium were applied before transplanting, and the remaining nitrogen dose was applied in two split doses, i.e., 30 and 45 days after transplanting. Harvesting of onion bulbs was done when the leaves turned yellow and more than 50 percent neckfall was observed. The bulbs were dug out along with their leaves and the tops were removed, leaving a 2 cm neck portion attached to the bulbs. Harvested bulbs were kept under shade for curing and then sorting before grading was done.

### 2.2. Morphometric and Yield-Related Traits

The data recorded were concerned the plant height (cm), no. of leaves/plant, bulb diameter (cm), bulb yield/plant, and total soluble solids (TSS, °Brix). The replicate-wise data of morphological traits of 10 selected plants were measured after 100 days of transplanting. The bulb yield of 10 plants was harvested separately from control and saline environments. Bulbs were inspected visually for rotting or sprouting damage. Equatorial bulb diameter (cm) of the same 10 plants was measured with the help of the digital Vernier Caliper. The TSS content of the selected bulbs was measured just after bulb harvesting with the help of a portable hand refractometer (Erma Inc., Tokyo, Japan) as °Brix (%) at 20 °C.

### 2.3. Physio-Biochemical and Ion Estimation

A total of 36 accessions were characterized under a saline environment, and out of these, 14 contrasting accessions were further selected for detailed studies of physiological, biochemical, and antioxidant enzymes, as well as ion contents. Out of the 14 accessions, 7 accessions (POS35, L-28, GJWO-1, POS36, L-744, POS37, POS38) had ≤20% yield reduction under saline conditions and were categorised as salt-tolerant. The other 7 accessions (ALR, GJWO3, KKR, L-652, Agrifound White, L-920, Bhima Dark Red) had ≥40% yield reduction and were categorized as salt-sensitive. These 14 accessions were again transplanted in microplots during the rabi season of 2020–21 for physiological, biochemical, and ionic analysis, and the package of practices and salinity treatments were followed as per the previous year’s (2019–20) experiment. 

#### 2.3.1. Physiological Traits

Physiological and biochemical traits were measured at 55 days after transplanting. The method given by Weatheraly [14] was used to estimate the relative water content (RWC). The leaf membrane thermostability index was estimated by using the electrolyte leakage percentage following the procedure of Dionisio-Sese and Tobita [15]. Photosynthesis rate (Pn), transpiration rate (E), and stomatal conductance (gS) of the 3rd fully expanded leaves were estimated using a portable photosynthetic system (Li 6800, Li-Cor Biosciences, Lincoln, NE, USA) following the method of Kumar et al. [16].

#### 2.3.2. Biochemical Traits

The method given by Bates et al. [17] was used to estimate the proline content. H_2_O_2_ content was calculated as per the method given by Loreto and Velikova [18]. MDA content was measured at 532 and 600 nm by using same the supernatant which was used for the estimation of H_2_O_2_ concentration [19]. A modified approach was followed for the extraction of antioxidant enzymes, superoxide dismutase (SOD), and ascorbate peroxidase (APX) [20]. Peroxidase (POX) was estimated as per the method suggested by Beauchamp and Fridovich [21]. One unit of APX corresponded to a change in O.D. of 1.0 per minute [22]. The POX activity was determined by using 1.0 mol of H_2_O_2_ per minute [23]. Based on the breakdown of H_2_O_2_ at 240 nm, the catalase (CAT) activity was measured for 1 min [24].

#### 2.3.3. Ion Concentration

Na^+^ and K^+^ contents of leaves, roots, and bulbs were determined at the time of harvesting using the flame photometer (Systronics Flame Photometer 128, Olathe, KS, USA) and estimated as mg g^−1^ of dry weight. 

### 2.4. Statistical Analysis

In the first experiment, five observations, i.e., plant height (cm), number of leaves/plant, equatorial bulb diameter (cm), bulb yield/plant (g), and TSS (°Brix) were recorded for preliminary screening. Onion accessions were categorized on the basis of bulb yield reduction (%) under salinity stress in comparison to the control conditions. Based on percent bulb yield reduction, seven tolerant (≤20%) and seven susceptible (≥40%) accessions were selected for a second experiment to determine the physio-biochemical basis of salinity tolerance.

In experiment 2, all the recorded parameters were put on a Microsoft excel sheet thematically and a test of normality for each parameter was performed through the Shapiro–Wilk test to comply with the assumptions of ANOVA and the appropriate transformation procedure was applied for violated parameters. Further, the approach of two-way analysis of variance (ANOVA) was applied for estimating the effect of genotypes and salinity, and their interaction and group comparison was made between tolerant and sensitive cultivars through contrast analysis using STAR statistical software [25]. The relative contribution of different traits in total genetic divergence of the onion genotypes in control and salinity stress conditions were quantified through the method proposed by Singh [26]. To find out the significant differences in various traits recorded in onion genotypes under salinity stress and control conditions, Duncan’s Multiple Range Test (DMRT) was performed using STAR statistical software [25]. To predict the bulb yield under salinity stress, a response equation was derived through a stepwise regression approach, and significantly associated (*p* > 0.005) morphological, physiological, and biochemical traits with bulb yield were also prioritized for conducting future studies on salinity stress tolerance in onions. 

## 3. Results 

### 3.1. Effects of Salinity on Selected Onion Accessions

The combined ANOVA of fourteen accessions characterized under normal and salt stress environments showed highly significant differences (*p* ≤ 0.01) for physiological parameters (SPAD, RWC, MSI, Pn, E and gS), biochemical parameters (Proline, H_2_O_2_, MDA, CAT, APX, SOD and POX), growth and bulb parameters (PH, NL, BD, BYP and TSS), and ionic concentrations (root Na^+^/K^+^, shoot Na^+^/K^+^ and bulb Na^+^/K^+^) (Table 3). The results indicated that the amount of variability present among the accessions would be helpful in the selection and utilization of promising salt-tolerant genotypes in onion breeding. Genotypes and environmental interactions were also found to be significant (*p* ≤ 0.01) for all the studied parameters, except NL, suggesting that most of the genotypes performed differentially in varying environmental conditions. Thus, the significance of variances for both genotypes and the interaction effect indicated that all the studied parameters were highly influenced by genotypes as well as the salt stress condition. 

### 3.2. Impact of Salt Stress on Growth and Bulb Parameters

Five growth and bulb parameters were selected for preliminary screening of thirty six onion accessions under normal and saline environments. Salinity stress significantly affects the plant height, bulb diameter, bulb yield per plant, and total soluble solids by 6.07%, 3.07%, 11.38%, 31.24%, and 8.34% compared to control condition, respectively. The percent bulb yield reduction ranged from 14.88% in POS35 to 62.86% in Bhima Dark Red (Table S1). On the basis of bulb yield reduction (%) due to salinity stress, the 36 accessions were classified into three groups. Seven accessions (POS35, L-28, GWO-1, POS36, L-744, POS37, and POS38) had less than 20% bulb yield reduction and were identified as salt-tolerant, whereas seven genotype accessions (ALR, GJWO 3, Kalyanpur Red Round (KKR), L-652, Agrifound White, L-920, Bhima Dark Red) with more than 40% yield reduction were classified as salt-sensitive (Table 4). Finally, we selected fourteen accessions, i.e., seven tolerant and seven sensitive, to observe the differential behavior in their physiological and biochemical traits under control and saline environments. Among the fourteen accessions, the bulb yield per plant under the saline environment was the highest in tolerant accessions with a reduction percentage ranging from 14.88 (POS35) to 19.76% (POS38), whereas a severe decline was recorded in salt sensitive accessions ranging from 42.48 (ALR) to 62.86% (Bhima Dark Red) (Table 5). The parameters such as PH, BD, and BYP were significantly reduced under salinity stress; however, TSS content was significantly increased in onion bulb though salinity treatment. Interestingly, a non-significant difference in numbers of leaves per plant (NOL) was observed under control and salinity conditions (Table 6). 

### 3.3. Physiological Responses

**Figure 1:**
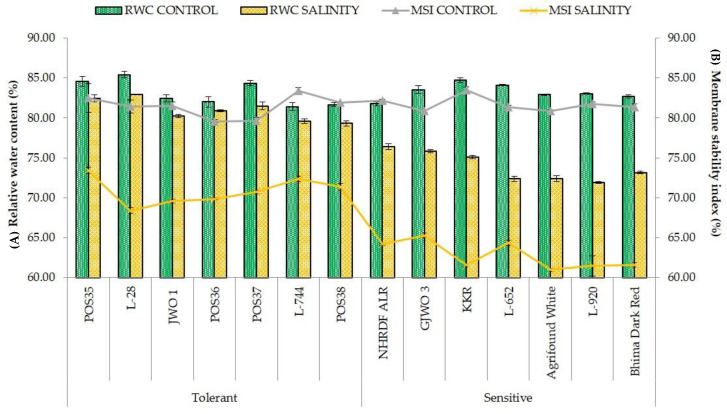
Changes in relative water content (%) (**A**); and membrane stability index (%) (**B**) of onion accessions under control and salinity stress.

**Figure 2:**
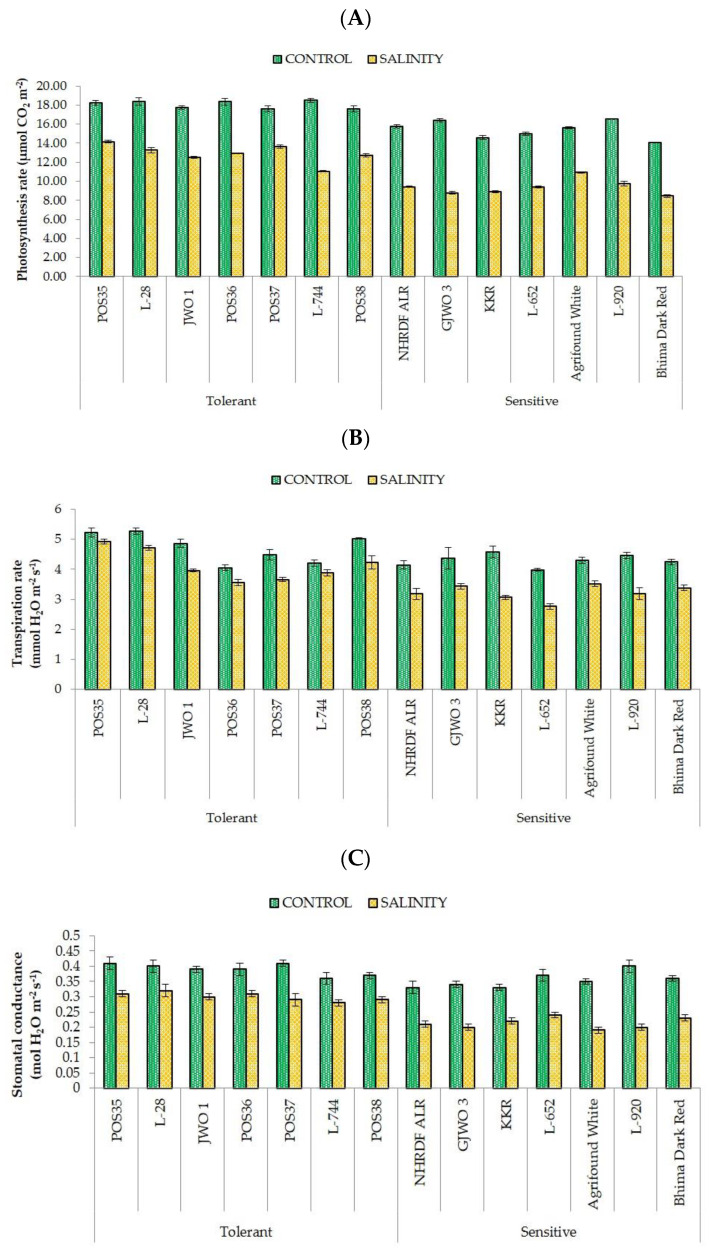
Alteration in gas exchange traits [Photosynthetic rate, (**A**); Transpiration rate, (**B**); and Stomatal conductance (**C**)] of onion accessions under control and saline environments.

Physiological parameters such as SPAD, RWC, MSI, Pn, E, and gS were found higher in tolerant accessions compared to sensitive ones under salt stress. RWC and MSI were related directly to salinity stress and vary significantly among all the onion accessions under salt stress. The tolerant accessions on average maintained higher RWC (>80%) and higher MSI (>70%) under stress conditions (Figure 1). Conversely, sensitive varieties showed lower RWC and more membrane damage. The percent reduction in RWC varied between 1.34 (POS36) to 3.41% (POS37) and 6.54 (ALR) to 13.96% (L-652) in tolerant and sensitive accessions, respectively. Likewise, the reduction percentage in MSI was 10.98 (POS35) to 16.02% (L-28) in tolerant and 19.27 (GJWO 3) to 26.24% (KRR) in sensitive varieties. A similar trend was observed for SPAD index. Tolerant varieties stayed green for a longer time whereas sensitive genotypes showed early leaf senescence in response to salt stress. Moreover, the photosynthetic rate and stomatal conductance were reduced significantly under a saline environment. The photosynthetic rate decreased by 28% in tolerant and by 39% in sensitive accessions. The highest Photosynthetic rate was observed in POS35 (14.17 μmol m^−2^ s^−1^) and POS37 (13.67 μmol m^−2^ s^−1^), whereas a minimum was found in Bhima Dark Red (8.45 μmol m^−2^ s^−1^) and GJWO 3 (8.80 μmol m^−2^ s^−1^). Furthermore, sensitive varieties showed a drastic reduction in stomata conductance (Figure 2) compared to tolerant ones. The maximum reduction percentage ranged from 18.52% (GJWO 1) to 29.27% (POS37) in salt-tolerant accessions and 33.19% (KRR) to 50% (L-920) in sensitive varieties, respectively. The tolerant cultivars maintained higher chlorophyll content, RWC, MSI, gS, and Pn compared to sensitive ones, except for the transpiration rate. 

### 3.4. Biochemical Responses

**Figure 3:**
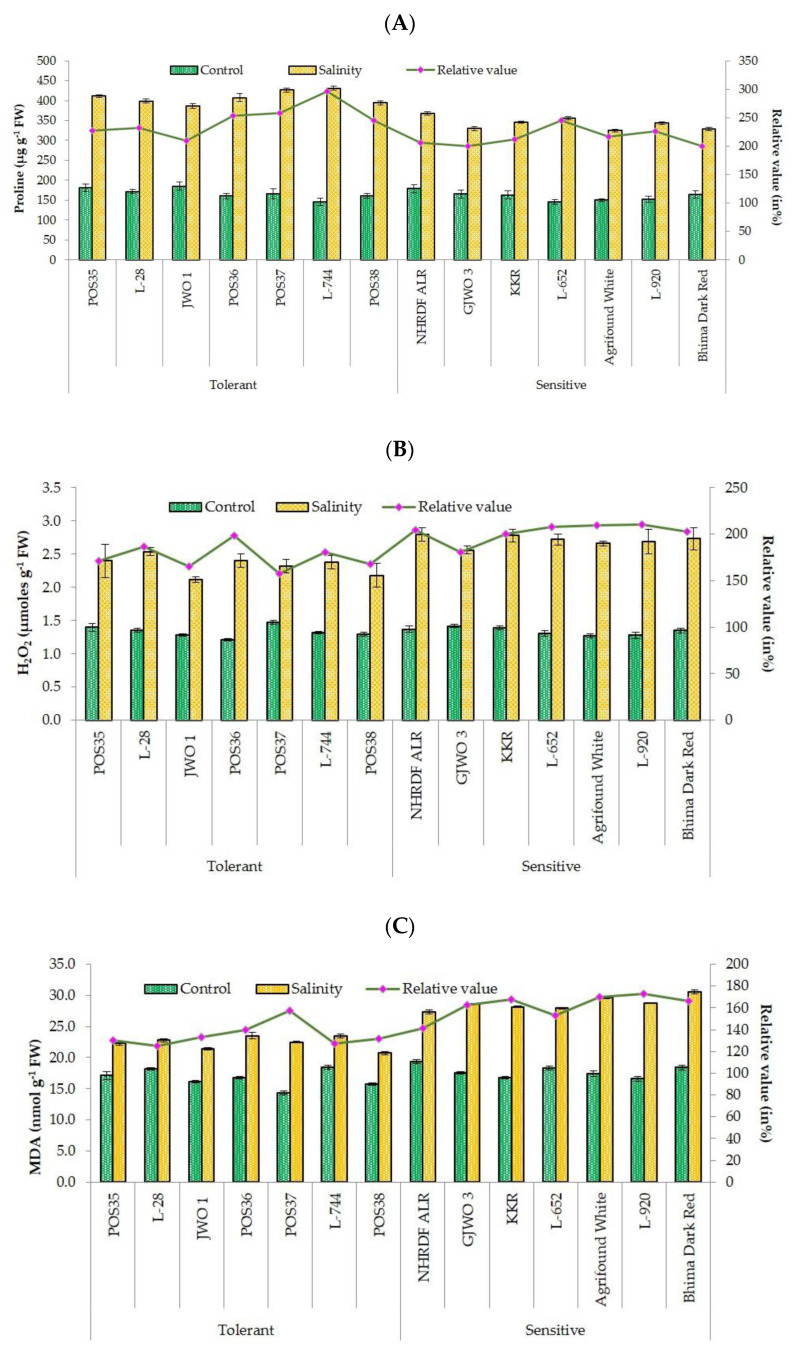
Alterations in biochemical parameters [Proline, (**A**); Hydrogen peroxide (H_2_O_2_) (**B**); and Malondialdehyde (MDA) (**C**)] of selected onion accessions under control and salinity stress.

**Figure 4:**
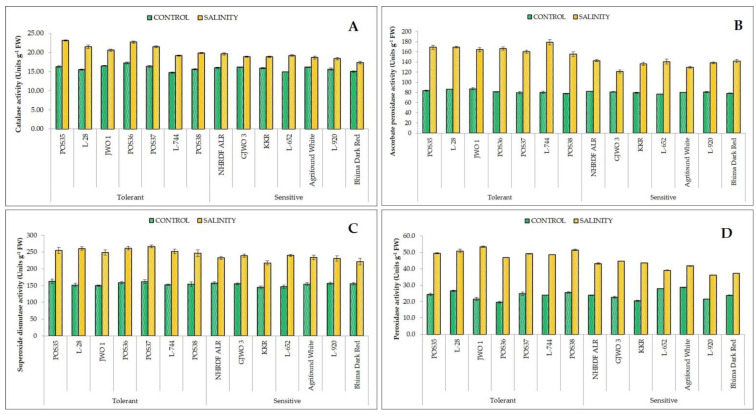
Antioxidative enzymes activity (CAT, (**A**); APX, (**B**); SOD, (**C**); and POX, (**D**)) of selected onion accessions under control and salinity.

Salinity stress significantly increased the production of H_2_O_2_ and lipid peroxidation via MDA content in all onion accessions (Figure 3). However, differential behaviors of salt tolerant and sensitive accessions were observed for H_2_O_2_ and MDA accumulation. The H_2_O_2_ content ranged from 2.56 µmoles/g FW (GJWO 3) to 2.80 µmoles/g FW (ALR) in salt-sensitive accessions, and from 2.12 µmoles/g FW (GJWO 1) to 2.54 µmoles/g FW (L-28) in salt-tolerant accessions under a saline environment. Similarly, the accumulation of MDA was higher in sensitive accessions, i.e., 27.33 nmol/g FW(ALR) to 30.60 nmol/g FW (Bhima Dark Red), with lower accumulation in tolerant ones, i.e., 20.74 nmol/g FW (POS38) to 23.50 nmol/g FW (POS36) under stress conditions. The activity of proline and antioxidant defense-related enzymes (CAT, APX, SOD, and POX), which are involved in ROS scavenging during stress conditions, were found to be higher in salt-tolerant and lower in salt-sensitive accessions. The maximum increment in proline content ranged from 109% (GJWO 1) to 196% (L-744) in salt-tolerant and 100% (GJWO 3) to 145% (L-652) in salt-sensitive accessions (Figure 3). Likewise, the percent increment in antioxidants activity of CAT, APX, SOD, and POX varied between 24.76 (GJWO 1) to 41.96% (POS35), 88 (GJWO 1) to 123% (L-744), 57 (POS35) to 72% (L-28), and 92.14 (L-28) to 149.53% (GJWO 1) in salt-tolerant accessions, whereas the range was 15.61 (Agrifound White) to 28.55% (L-652), 49 (GJWO 3) to 83% (L-652), 42 (Bhima Dark Red) to 64% (L-652), and 39.64 (L-652) to 113.52% (KKR) in salt-sensitive accessions, respectively (Figure 4). The tolerant cultivars show a higher activity of antioxidant enzymes and lower production of H_2_O_2_ and lipid peroxidation compared to sensitive ones (Table 6).

### 3.5. Accumulation of Ion Concentrations

Under a saline environment, Na^+^ accumulation increased significantly in the roots, shoots, and bulbs of all the fourteen onion accessions (Table 6). The Na^+^/K^+^ ratio in the roots ranged from 1.05 (L-920) to 2.48 (KRR) under control and from 3.10 (GJWO 1, POS 36) to 3.79 (KRR) under stress conditions. In shoots, the range varied between 0.71 (L-920) to 1.67 (POS 36) under control and 1.44 (POS37) to 3.88 (ALR) under salinity stress. Similarly, the ratio in bulbs was 0.19 (Bhima Dark Red and Agrifound White) to 0.26 (GJWO 1 and ALR) and 0.26 (POS 36) to 0.41 (GJWO1) under control and stress conditions, respectively. The increase in the Na^+^/K^+^ ratio was found more in salt-sensitive accessions than in salt-tolerant ones (Table 6). The percent increment in roots, shoots, and bulbs ranged from 53.70 (POS38) to 169.57% (GJWO 1), 35.85 (POS37) to 130.48% (GJWO 1), 13.04 (POS 36) to 57.69% (GJWO 1) in salt-tolerant accessions, and 47.42 (Bhima Dark Red) to 213.33% (L-920), 128.30 (L-652) to 288.73% (L-920), and 19.23 (ALR) to 94.74% (Bhima Dark Red) in salt-sensitive varieties, respectively (Table S2).

### 3.6. Traits Contributing towards Bulb Yield Divergence

The percentage change in twenty traits in the saline environment and their direction of magnitude are represented in (Table 7). PRO (129.33%), H_2_O_2_ (88.38%), MDA (48.27%), CAT (25.90%), APX (86.18%), SOD (57.52%), POX (90.01%), Root Na^+^/K^+^ (102.54%), Shoot Na^+^/K^+^ (136.61%), and Bulb Na^+^/K^+^ (41.54%) showed an increment, whereas SPAD (4.15%), RWC (6.92%), MSI (18.09%), Pn (33.42%), E (18.53%), gS (31.09%), PH (5.45%), NL (1.79%), BD (12.04%), BYP (30.01%), and TSS (8.30 °Brix) showed a decrease in the mean value in the saline environment. Divergence analysis revealed that BYP (73.66%) followed by TSS (6.19 °Brix) and Shoot Na^+^/K^+^ (5.79%) were the greatest toward genetic divergence of the fourteen accessions under control conditions, whereas BYP (78.80%), followed by POX (3.83%), MDA (3.27%), RWC (2.58%), and Shoot Na^+^/K^+^ (2.54%) contributed maximally under salt stress. (Table 8) Thus, these traits can be exploited for the identification of genetically divergent parents for the genetic improvement program of onions. A stepwise regression analysis was done to identify the component variables contributing significantly to bulb yield under salinity stress. The results indicated that BD, PH, APX, gS, POX, CAT, MDA, MSI, and bulb Na^+^/K^+^ ratio accounted for the maximum variation of bulb yield in onions under stress conditions with cumulative R^2^ = 97.60. A significantly positive regression coefficient of BD, PH, APX, POX, MDA, MSI, and bulb Na^+^/K^+^ ratio indicated that an increment in the value of these traits might increase the bulb yield of onions. On the basis of regression coefficients of significant traits, the predicted model equation for bulb yield under salt stress (Table S3) was computed as:

Predicted bulb yield = −409.75 + (21.38 × BD) + (2.28 × PH) + (0.52 × APX) + (−137.77 × gS) + (1.21 × POX) + (−1.47 × CAT) + (3.21 × MDA) + (1.44 × MSI) + (Bulb Na^+^/K^+^)

## 4. Discussion

To study the physiological and biochemical responses of thirty six onion accessions, we first evaluated them based on growth and bulb parameters. The results of the primary trial showed a significant reduction in plant height (1.07–12.91%), bulb diameter (0.37–25.85%), bulb yield per plant (14.88–62.86%), and total soluble solids (1.43–20%) under salinity stress in comparison to control (Table S1). However, accessions differences were observed for reduction percentage in studied parameters. Based on percent bulb yield reduction, we selected seven salt-tolerant (less than 20% yield reduction) and seven-salt sensitive (more than 40% yield reduction) accessions to study the various physiological and biochemical changes in onion varieties as adaptive mechanisms. Plant height reduction in onions may be due to a decline in cell division and cell expansion under stress conditions, which ultimately reduces the overall plant growth [27]. Moreover, refs. [28,29] reported that increased salt stress reduces leaf growth, number of leaves, number of branches, and stem diameter. Onions are a salt-sensitive crop and irrigation with saline water reduces the production of large sized bulbs, fresh weight, bulb firmness, water use efficiency, and bulb yield [11]. Furthermore, a differential reduction rate in water dropwort cultivars for plant height, number of branches, number of leaves, and stem length was observed with increasing salt concentrations [30]. Accumulation of excessive salt in leaves might be the probable cause of reduction in the leaf area of onions [31,32,33].

RWC and MSI are the most commonly used adaptive parameters for the selection of tolerant accessions under abiotic stress environments. Maintenance of high RWC and MSI are decisive indicators of high cellular turgidity and less cellular or membrane injuries in plants [33]. Additionally, most of the physiological and biochemical processes such as cell division, cell enlargement, stomatal opening, and transportation in plants are dependent on the water status of the plant cells. Therefore, any reduction in water status adversely affects the plant’s metabolic functioning. Our results demonstrated that tolerant onion varieties showed high RWC and MSI rather than sensitive genotypes showing an agreement with the results of [34,35,36]. Furthermore, the ability of plants to maintain a normal photosynthetic rate, stomatal conductance, and transpiration rate reflects the ability of salt tolerance. The obtained results in the present study showed a negative effect of salt stress on SPAD, Pn, gS, and E in all fourteen accessions. However, tolerant accessions showed a lesser reduction percentage compared to sensitive ones. These results are in agreement with the findings of earlier studies [37,38,39] that reported a significant decrease in photosynthetic rate, transpiration rate, and chlorophyll content under salt stress. The decline in SPAD index under salinity may be due to photo-oxidation, loss of chloroplast membranes, and membrane injury in chloroplasts by ROS [40,41,42]. However, in some studies, higher chlorophyll content was recorded in the saline environment, which may have been caused by the increase in number of chloroplasts [30,43]. Moreover, under excessive salinity, fewer gaseous exchanges occur in leaves because of the high sensitivity of stomata to abiotic stresses, leading to a reduction in transpiration and photosynthetic rates [44,45,46,47]. Lipid peroxidation (MDA) and production of reactive oxygen species (H_2_O_2_) are commonly used biochemical indicators for plants exposed to abiotic stresses [48]. The high concentration of MDA and H_2_O_2_ under salt stress leads to cell membrane damage and electrolyte leakage [49]. In the present research, the accumulation of MDA and H_2_O_2_ was less in tolerant accessions compared to sensitive ones, indicating less oxidative damage to cell membranes of tolerant accessions. These results are also supported by other studies [30,50,51], in which lower ROS production and lipid peroxidation and high membrane stability in salt-tolerant genotypes of rice, wheat, and water dropwort were reported. The compatible solute proline plays a very important role in maintaining the osmotic potential of cytosol with the external environment [52]. The proline activity was observed as high under salt-stressed plants of tolerant accessions, whereas in sensitive accessions, the activity was comparatively lower. The enhanced activity of proline might be the important factor in neutralizing the negative effect of salinity stress via osmotic adjustment [53]. Furthermore, a greater activity of antioxidants (SOD, APX, CAT, and POX) provides salt tolerance by scavenging ROS and reducing membrane damage [54]. In defensive response, the SOD and CAT convert free radical species into H_2_O_2_ and oxygen, respectively [55]. Salt tolerance in tomato, potato, cabbage, and amaranth caused by higher antioxidants activity were also reported [48,56,57,58,59]. A positive correlation between enhanced antioxidant activity and reduced oxidative damage has been observed in the present study. The tolerant varieties under salt stress had better activity of these enzymes than those of the sensitive varieties.

Higher salt accumulation in the rhizosphere of plants leads to the accumulation of Na^+^ and Cl^−^ ions, causing an osmotic effect, ionic imbalance, damage to enzymatic activities, and protein metabolism [60]. Ionic imbalance causes a reduced uptake of essential nutrients such as potassium, manganese, and calcium to plant cells. The increased uptake of external Na^+^ will enhance the Na^+^ concentration in different plant organs with a concomitant decrease in K^+^ [12,48,61]. However, under high salinity stress, tolerant plants have a unique ability to accumulate and compartmentalize Na^+^ and Cl^−^ in older leaves, but sensitive accessions cannot manage such compartmentalization, which leads to ionic and osmotic effects [61]. This compartmentalization of Na^+^ into vacuoles is controlled by Na^+^/H^+^ antiporters, V-type H^+^-ATPase, and H^+^-PPase [62]. In this study, a higher Na^+^/K^+^ ratio was found in the roots, shoots, and bulbs, but percentage increase in the ratio was higher in sensitive accessions compared to tolerant accessions. Previous studies in carrot, amaranth, and pistachio also showed an increased uptake of Na^+^ and decreased uptake of K^+^ in salt-sensitive genotypes under salinity stress [7,51,63]. Therefore, many authors suggested that maximal uptake of K^+^ and minimal uptake of Na^+^ is an indicator of salt tolerance in crop plants [30,51].

Stepwise regression analysis is a statistical method which identifies the most important contributing variable, signifying the amount of variability towards dependent variables such as economic yield. Based on this approach, we identified bulb diameter, plant height, ascorbate peroxidase, stomatal conductance, peroxidase, catalase, malondialdehyde, membrane stability index, and bulb Na^+^/K^+^ ratio as significantly contributing traits towards the total variation present in bulb yield per onion plant under salt stress. The significantly positive and negative coefficient of traits indicates that with increase or decrease of their respective value, there will be an increase or decrease in the final bulb yield per plant, respectively. In the previous research, Bojarian et al. [64] also reported that single fruit weight, diameter, pericarp thickness, and titratable acidity are the important traits for successful breeding programs in tomatoes. Similarly, Saed-Moucheshi et al. [65] selected spike weight and chlorophyll content as major traits for wheat breeding programs under different water regime conditions.

## 5. Conclusions

A wide range of variability was observed in studied onion genotypes for bulb yield and yield associated with morphological, physiological, and biochemical traits. The tolerant accessions selected based on percent yield reduction had strong antioxidant defense systems and lower Na^+^/K^+^ ratios in the shoot/leaves and maintained a higher tissue water status and osmoprotectants. Statistical analysis indicated that salinity stress significantly and negatively affects the bulb yield and yield-associated traits in onions, except number of leaves per plant. Interestingly, the salt-tolerant onion-tolerant cultivars showed a higher activity of antioxidant enzymes (APX, SOD, POX, and CAT), higher chlorophyll content (RWC, MSI, gS, and Pn), and lower production of H_2_O_2_ and lipid peroxidation compared to sensitive ones. Furthermore, the performance of onion cultivars assessed through traits modeling indicated a total of nine morphological, physiological, and biochemical traits (BD, PH, APX, gS, POX, CAT, MDA, MSI, and bulb Na^+^/K^+^ ratio), accounting for maximum variation in bulb yield under salinity stress, and which are the highly weighted variables that can be utilized for onion germplasm screening for salinity stress tolerance. From the experimental findings, we could summarize that the onion cultivars POS35, NHRDF Red (L-28), GWO 1, POS36, NHRDF Red-4 (L-744), POS37, and POS38 may be considered as saline-tolerant. These selected cultivars could be directly recommended for enhancing agricultural resilience in saline agro-ecosystems and can be utilized as potential genetic resources (salt-tolerant donor parents) in onion improvement programs.

## Figures and Tables

**Table 1:** Passport information of onion accessions collected for salinity tolerance evaluation.

S. No.	Varieties	Parentage	Year of Release	Maturity (Days)	Bulb Color	Bulb Shape	Bulb Size	Storability (Months)
1.	Pusa Red	Mass Selection (Local collection)	1975	125–140	Bronze red	Flat to globular	Medium	3–4
2.	Kalyanpur Red Round (KKR)	Mass Selection (Collection from UP)	1983	110–120	Red	Round	Medium	3–4
3.	ALR	Mass Selection (Collection from Nasik, Maharashtra)	1988	110–120	Light red	Globular	Medium	3–4
4.	B-780 (Baswant780)	Mass Selection (Collection from Pimpalgaon, Maharashtra)	1989	110–120	Dark red	Round	Large	2–3
5.	Agrifound White	Mass Selection (Local Collection)	1994	160–165	White	Round	Medium	1–2
6.	Punjab Naroya	Mass selection of material collected from Maharashtra	1997	115–120	Red	Round	Medium to large	2–3
7.	GJWO-1	Mass Selection (Collection from Gujarat)	2000	130–135	White	Round	Medium	2–3
8.	RO 59 (Arpita)	Mass Selection (Local Collection)	2005	110–120	Red	Globe	Medium	2–3
9.	Arka Pitambar	Selection from U.D. 102 × IAR-396	2006	135–140	Yellow	Globe	Medium	3–4
10.	NHRDF Red (L-28)	Collected from Patiala, Punjab and developed at RRS, Karnal.	2006	180–210	Dark red	Round	Medium	2–3
11.	Bhima Super	Rigorous mass selection for single centeredness and bulb shape	2006	100–110	Red	Flat globe	Large	2–3
12.	Bhima Raj	Single bulb selection up to three generations	2007	120–125	Red	Oval	Medium	2–3
13.	Bhima Red	Single bulb selection up to three generations	2009	115–120	Red	Round	Medium	2–3
14.	Bhima Shweta	(White El. Comp. Selection/NRCWO2) (IC No. 572761)	2010	110–120	White	Round	Medium	2–3
15.	Bhima Shakti	IC No. 572769	2010	125–135	Red	Round	Large	5–6
16.	POS38	Selfing (two generations) and massing from Bhima Kiran	2010	125–135	Light red	Oval to round	Large	5–6
17.	Arka Bheem	Triparental synthetic	2011	125–130	Red to pinkish red	Elongated globe	Medium	1–2
18.	RO-252	Mass Selection (Local Collection)	2011	-	Red	-	Medium	2–3
19.	Bhima Dark Red	Single bulb selection up to three generations	2012	100–110	Dark red	Flat round	Medium	1–1.5
20.	NHRDF Red-3 (L-652)	Mass Selection (Local Collection)	2012	100–120	Bronze red	Globular round	Medium	2–3
21.	Bhima Safed	Mass Selection (Local Collection)	2014	110–120	White	Round to oval	Medium	1–1.5
22.	JRO 11 (GJRO 11)	Local collection from Mahuva Taluka of Bhavnagar district	2015	125–130	Red	Flat globe	Medium	3–4
23.	Bhima Light Red	Single bulb selection followed by mass selection	2015	115–120	Light red	Globe	Large	2–3
24.	HOS 4 (Hisar onion 4)	Selection from local material collected from Bahadurgarh area	2016	130–140	Light Bronze	Globular	Medium	3–4
25.	GJWO 3	Local collection from Talaja Taluka of Bhavnagar district Germplasm No. 120	2016	125–130	White	Flat globe	Medium	2–3
26.	NHRDF Red-4 (L-744)	Mass Selection (Local Collection)	2016	110–120	Red	Globular round	Medium	2–3
27.	Pusa Shobha	Mass selection from segregating material from local collection	2018	140–160	Brown	Flat globe	Large	1–2
28.	Pusa Sona	Mass selection from segregating material of Early Grano	2019	125–135	Creamy yellow	Globular	Large	1–2
29.	POS35	Selection-Selfing (two generations) followed by massing from Pusa White Round selection	-	125–130	White	Round	Medium	2–3
30.	POS36	Selection- Selfing (two generations) followed by massing from Pusa Madavi	-	-	Brown	Globe	Large	2–3
31.	POS39	Single plant selection from Hisar 2 with waxy leaves (Hisar2 Waxy)	-	125–130	Bronze red	Flat globular	Medium	3–4
32.	POS37	Selfing (two generations) followed by massing from Pusa White Flat	-	120–130	White	Flat round	Medium large	2–3
33.	Sukhsagar	Local landrace from West Bengal	-	90–100	Dark red	Globe	Medium	3–4
34.	PRO 6	Mass Selection (Collection from Punjab)		115–120	Deep red	Round	Medium large	2–3
35.	JNDWO-85	Mass Selection (Collection from Gujarat)	-	-	White	-	-	3–4
36.	NHRDF (L-920)	Mass Selection (Local Collection)	-	-	-	-	Medium	2–3

**Table 2:** Soil status: Initial and final soil salinity.

Parameters	Initial Soil Status	Final Soil Status	
		Control	After Saline Treatment
2019–20			
ECe (dS m^−1^)	1.65	1.85	6.90
pHs	7.74	7.67	7.82
2020–21			
ECe (dS m^−1^)	1.48	1.62	7.04
pHs	7.61	7.65	7.93

**Table 3:** Variance analysis of morpho-physiological and biochemical traits of 14 selected onion accessions over control and saline environment.

Variables	Mean Squares		F Values		Significance	
	Genotypes	G X E	Genotypes	G X E	Genotypes	G X E
**Df**	**13**	**13**	**13**	**13**	**Pr(>F)**	**Pr(>F)**
^#^SPAD Index	21.66	3.26	54.97	8.26	0.000	0.000
RWC	28.51	24.69	233.79	202.46	0.000	0.000
MSI	31.88	32.85	112.93	116.38	0.000	0.000
Pn	17.51	1.88	451.38	48.55	0.000	0.000
E	1.49	0.19	83.42	10.41	0.000	0.000
gS	0.01	0.00	36.77	9.25	0.000	0.000
Proline	2596.14	2045.07	47.65	37.54	0.000	0.000
H_2_O_2_	0.09	0.08	9.44	8.25	0.000	0.000
MDA	26.56	13.90	315.53	165.17	0.000	0.000
CAT	7.22	2.89	154.75	61.89	0.000	0.000
APX	524.63	403.66	89.91	69.18	0.000	0.000
SOD	481.60	279.97	15.71	9.13	0.000	0.000
POX	53.36	58.10	287.12	312.58	0.000	0.000
Root Na^+^/K^+^	0.51	0.20	51.19	20.36	0.000	0.000
Shoot Na^+^/K^+^	1.50	0.92	578.09	354.26	0.000	0.000
Bulb Na^+^/K^+^	0.00	0.00	9.74	8.97	0.000	0.000
PH	31.05	3.86	82.25	10.23	0.000	0.000
NL	0.82	0.02	44.17	1.01	0.000	0.453
BD	1.64	0.18	86.95	9.28	0.000	0.000
BYP	1361.39	134.57	11990.75	1185.22	0.000	0.000
TSS	6.40	3.28	284.75	146.11	0.000	0.000

^#^SPAD index: Soil Plant Analysis Development Chlorophyll Meter index, RWC: Relative Water Content, MSI: Membrane Stability Index, Pn: Photosynthesis rate, E: Transpiration rate, gS: Stomatal conductance, H_2_O_2_: Hydrogen peroxide), MDA: Malondialdehyde, CAT: Catalase, APX: Ascorbate peroxidase, SOD: Superoxide dismutase, (POX: Peroxidase, PH: Plant height, NL: Number of leaves/plant, BD: Bulb diameter, BYP: Bulb yield/plant, TSS: Total soluble solids.

**Table 4:** Grouping of onion accessions on % bulb yield reduction in saline environment.

Salt-Tolerant Genotypes	Bulb Yield Reduction (<20%)	Moderately Tolerant Genotypes	Bulb Yield Reduction (20–40%)	Salt-Sensitive Genotypes	Bulb Yield Reduction (>40%)
POS35	14.88	Bhima Red	20.20	ALR	42.48
L-28	15.13	POS39	20.55	GJWO 3	42.63
GJWO 1	16.35	Bhima Shweta	24.72	KKR	43.50
POS36	17.19	JNDWO-85	26.65	L-652	43.65
L-744	18.80	HOS 4 (Hisar onion 4)	29.43	Agrifound White	43.89
POS37	19.48	Arka Pitamber	29.52	L-920	48.60
POS38	19.76	Pusa Red	29.56	Bhima Dark Red	62.86
		RO 59 (Arpita)	29.99		
		Pusa Shobha	31.47		
		Bhima Light Red	32.15		
		Punjab Naroya	32.31		
		GJRO 11	32.92		
		Bhima Raj	34.60		
		PRO 6	34.60		
		B-780 (Baswant780)	34.69		
		Bhima Shakti	34.96		
		Bhima Super	35.25		
		Bhima Safed	35.27		
		RO-252	37.11		
		Sukhsagar	38.22		
		Pusa Sona	38.44		
		Arka Bheem	39.62		

**Table 5:** Means comparisons of morphological traits of selected onion accessions under control and salinity stress.

Cultivars Name	Plant Height (cm)		Number of Leaves		Bulb Diameter (cm)		Bulb Yield (g plant^−1^)		TSS (°Brix)	
	Control	Salinity	Control	Salinity	Control	Salinity	Control	Salinity	Control	Salinity
POS35	43.22 ± 0.31 *^de^*	42.20 ± 0.43 *^de^*	8.30 ± 0.04	8.27 ± 0.25	6.18 ± 0.04 *^a^*	5.62 ± 0.13 *^a^*	77.48 ± 0.59 *^a^*	65.95 ± 0.05 *^a^*	12.43 ± 0.03 *^e^*	12.00 ± 0.06 *^d^*
L-28	43.95 ± 0.64 *^cd^*	43.48 ± 0.73 *^abc^*	9.01 ± 0.11	8.98 ± 0.08	5.80 ± 0.08 *^b^*	5.44 ± 0.10 *^ab^*	77.00 ± 0.20 *^a^*	65.35 ± 0.35 *^b^*	12.07 ± 0.07 *^g^*	10.67 ± 0.13 *^g^*
GJWO 1	43.82 ± 0.43 *^d^*	42.62 ± 0.81 *^bcd^*	8.17 ± 0.24	7.96 ± 0.06	5.31 ± 0.04 *^cd^*	4.94 ± 0.06 *^c^*	58.70 ± 0.30 *^g^*	49.10 ± 0.10 *^f^*	12.37 ± 0.10 *^ef^*	10.37 ± 0.07 *^h^*
POS36	43.25 ± 0.78 *^de^*	42.26 ± 0.55 *^de^*	8.35 ± 0.10	8.15 ± 0.13	5.87 ± 0.13 *^b^*	5.02 ± 0.08 *^c^*	66.95 ± 0.20 *^f^*	55.44 ± 0.44 *^e^*	13.37 ± 0.10 *^e^*	12.30 ± 0.30 *^c^*
POS37	43.20 ± 0.40 *^de^*	42.55 ± 0.44 *^cde^*	8.70 ± 0.15	8.48 ± 0.29	5.69 ± 0.52 *^b^*	5.54 ± 0.07 *^a^*	70.65 ± 0.15 *^d^*	56.89 ± 0.11 *^d^*	12.77 ± 0.10 *^d^*	12.07 ± 0.07 *^cd^*
L-744	45.43 ± 0.58 *^ab^*	44.03 ± 0.62 *^a^*	8.85 ± 0.05	8.71 ± 0.10	5.92 ± 0.10 *^b^*	5.29 ± 0.10 *^b^*	74.38 ± 1.20 *^b^*	60.40 ± 0.40 *^c^*	12.17 ± 0.17 *^fg^*	11.63 ± 0.20 *^e^*
POS38	38.75 ± 0.32 *^h^*	37.65 ± 0.78 *^g^*	9.06 ± 0.11	8.91 ± 0.09	5.28 ± 0.14 *^cd^*	4.87 ± 0.07 *^c^*	69.67 ± 0.10 *^e^*	55.90 ± 0.10 *^e^*	12.10 ± 0.10 *^g^*	11.50 ± 0.20 *^e^*
ALR	44.88 ± 1.18 *^b^^c^*	41.45 ± 0.69 *^e^*	8.76 ± 0.10	8.56 ± 0.20	4.70 ± 0.10 *^e^*	4.17 ± 0.13 *^ef^*	41.53 ± 0.10 *^k^*	23.89 ± 0.11 *^j^*	12.90 ± 0.10 *^d^*	12.20 ± 0.20 *^cd^*
GJWO 3	41.93 ± 0.60 *^f^*	37.46 ± 0.55 *^g^*	7.99 ± 0.06	7.84 ± 0.16	5.38 ± 0.06 *^c^*	4.21 ± 0.09 *^def^*	49.30 ± 0.30 *^j^*	28.28 ± 0.28 *^h^*	12.50 ± 0.10 *^e^*	14.85 ± 0.06 *^a^*
KKR	42.25 ± 0.73 *^ef^*	37.65 ± 0.78 *^g^*	7.90 ± 0.08	7.87 ± 0.20	5.06 ± 0.04 *^d^*	4.43 ± 0.13 *^d^*	40.00 ± 0.25 *^l^*	22.60 ± 0.60 *^k^*	15.77 ± 0.10 *^b^*	11.00 ± 0.15 *^f^*
L-652	42.37 ± 0.65 *^ef^*	39.82 ± 0.83 *^f^*	8.45 ± 0.15	8.30 ± 0.09	5.26 ± 0.11 *^cd^*	4.32 ± 0.12 *^de^*	55.10 ± 0.10 *^h^*	31.05 ± 0.05 *^g^*	12.93 ± 0.07 *^d^*	12.07 ± 0.07 *^cd^*
Agrifound White	40.66 ± 0.62 *^g^*	35.49 ± 1.06 *^h^*	8.25 ± 0.05	8.10 ± 0.09	5.10 ± 0.06 *^d^*	4.86 ± 0.06 *^c^*	50.22 ± 0.22 *^i^*	28.18 ± 0.18 *^h^*	13.60 ± 0.30 *^c^*	11.97 ± 0.03 *^d^*
L-920	46.42 ± 0.56 *^a^*	43.67 ± 0.90 *^ab^*	8.08 ± 0.08	8.06 ± 0.06	4.72 ± 0.06 *^e^*	3.50 ± 0.06 *^g^*	41.56 ± 0.20 *^k^*	21.36 ± 0.36 *^l^*	16.10 ± 0.05 *^a^*	14.30 ± 0.30 *^b^*
Bhima Dark Red	45.26 ± 0.75 *^b^*	42.08 ± 0.35 *^de^*	8.74 ± 0.14	8.29 ± 0.07	5.09 ± 0.09 *^d^*	4.06 ± 0.08 *^f^*	72.10 ± 0.10 *^c^*	26.78 ± 0.10 *^i^*	12.17 ± 0.17 *^fg^*	11.10 ± 0.10 *^f^*

Numerical values are represented as Mean ± SD; means comparison was performed through Duncan’s Multiple Range Test (DMRT). Values with the same letter are not significantly different.

**Table 6:** Effects of salinity stress on tolerant and sensitive onion cultivars through group comparison analysis.

Trait	Unit	Groups Mean		Mean Square	Significance
		Tolerant Genotypes	Sensitive Genotypes	Tolerant vs. Sensitive	Pr(>F)
SPAD Index	Value	44.90 ± 1.40 *^a^*	42.29 ± 2.05 *^b^*	33.571	0.000
Relative Water Content (RWC)	%	80.98 ± 1.36 *^a^*	73.87 ± 1.77 *^b^*	116.433	0.000
Membrane Stability Index (MSI)	%	70.81 ± 1.66 *^a^*	62.76 ± 1.72 *^b^*	215.15	0.000
Photosynthesis rate (Pn)	µmol CO_2_ m^−2^ s^−1^	12.91 ± 0.95 *^a^*	9.38 ± 0.79 *^b^*	8.397	0.000
Transpiration rate (E)	mmol H_2_O m^−2^ s^−1^	4.13 ± 0.50 *^a^*	3.22 ± 0.27 *^a^*	0.024	0.160
Stomatal conductance (gS)	mol H_2_O m^−2^ s^−1^	0.30 ± 0.02 *^a^*	0.21 ± 0.02 *^b^*	0.006	0.000
Proline content (Pro)	µg g^−1^ FW	407.88 ± 17.29 *^a^*	342.62 ± 16.77 *^b^*	11853.07	0.000
Hydrogen peroxide (H_2_O_2_)	µmoles g^−1^ FW	2.33 ± 0.18 *^b^*	2.71 ± 0.12 *^a^*	0.666	0.000
Malondialdehyde (MDA)	nmol g^−1^ FW	22.38 ± 0.99 *^b^*	28.70 ± 1.06 *^a^*	108.58	0.000
Catalase (CAT)	units g^−1^ FW	21.18 ± 1.39 *^a^*	18.68 ± 0.72 *^b^*	5.838	0.000
Ascorbate peroxidase (APX)	units g^−1^ FW	166.11 ± 7.70 *^a^*	135.77 ± 7.75 *^b^*	870.59	0.000
Super oxide dismutase (SOD)	units g^−1^ FW	255.76 ± 9.20 *^a^*	230.75 ± 9.70 *^b^*	1687.96	0.000
Peroxidase (POX)	units g^−1^ FW	49.96 ± 2.07 *^a^*	40.78 ± 3.18 *^b^*	333.26	0.000
Root Na^+^/K^+^	Index	3.22 ± 0.13 *^b^*	3.50 ± 0.25 *^a^*	0.367	0.000
Shoot Na^+^/K^+^	Index	2.11 ± 0.43 *^b^*	3.57 ± 0.37 *^a^*	1.765	0.000
Bulb Na^+^/K^+^	Index	0.32 ± 0.05 *^b^*	0.34 ± 0.04 *^a^*	0.004	0.007
Plant height (PH)	Cm	41.82 ± 1.97 *^a^*	39.95 ± 3.12 *^b^*	8.52	0.000
Number of leaves/plant (NL)	Nos.	8.43 ± 0.35 *^a^*	8.21 ± 0.38 *^a^*	0.065	0.101
Bulb diameter (BD)	Cm	5.07 ± 0.47 *^a^*	4.40 ± 0.59 *^b^*	0.138	0.001
Total soluble solids (TSS)	°Brix	11.72 ± 0.65 *^b^*	12.28 ± 1.58 *^a^*	2.024	0.000
Bulb yield/plant (BYP)	g plant^−1^	52.51 ± 12.92 *^a^*	31.94 ± 14.34 *^b^*	52.371	0.000

Numerical values are represented as Mean ± SD, and values between the column with the same letter are not significantly different.

**Table 7:** Relative contribution of different physiological, biochemical, and yield-related traits toward genetic divergence in onion accessions.

Traits	Traits Contribution (%) under Control Conditions	Traits Contribution (%) under Salinity Stress Conditions	Traits Mean ± SD		Magnitude # (%)
			Control	Salinity Stress	
SPAD Index	0.414	0.293	45.49 ± 0.49	43.60 ± 0.34	−4.15
Relative Water Content (RWC)	0.707	2.580	83.18 ± 0.33	77.43 ± 0.26	−6.92
Membrane Stability Index (MSI)	0.227	1.617	81.54 ± 0.43	66.79 ± 0.35	−18.09
Photosynthesis rate (Pn)	2.637	3.018	16.74 ± 0.21	11.15 ± 0.14	−33.42
Transpiration rate (E)	0.478	0.484	4.51 ± 0.13	3.67 ± 0.11	−18.53
Stomatal conductance (gS)	0.191	0.256	0.37 ± 0.02	0.26 ± 0.01	−31.09
Proline content (Pro)	0.374	0.256	163.63 ± 4.75	375.25 ± 8.42	+129.33
Hydrogen peroxide (H_2_O_2_)	0.263	0.043	1.34 ± 0.03	2.52 ± 0.11	+88.38
Malondialdehyde (MDA)	0.960	3.266	17.23 ± 0.30	25.54 ± 0.22	+48.27
Catalase (CAT)	1.423	0.607	15.83 ± 0.18	19.93 ± 0.25	+25.90
Ascorbate peroxidase (APX)	0.498	0.430	81.07 ± 0.98	150.94 ± 3.24	+86.18
Super oxide dismutase (SOD)	0.124	0.072	154.42 ± 4.25	243.25 ± 6.48	+57.52
Peroxidase (POX)	1.811	3.832	23.88 ± 0.43	45.37 ± 0.30	+90.01
Root Na^+^/K^+^	1.878	0.058	1.66 ± 0.07	3.36 ± 0.10	+102.54
Shoot Na^+^/K^+^	5.786	2.536	1.20 ± 0.05	2.84 ± 0.07	+136.61
Bulb Na^+^/K^+^	0.120	0.058	0.23 ± 0.02	0.33 ± 0.02	+41.54
Plant height (PH)	1.140	0.217	43.24 ± 0.61	40.89 ± 0.68	−5.45
Number of leaves/plant (NL)	0.653	0.088	8.47 ± 0.10	8.32 ± 0.13	−1.79
Bulb diameter (BD)	0.462	0.640	5.38 ± 0.11	4.73 ± 0.09	−12.04
Bulb yield/plant (BYP)	73.662	78.800	60.33 ± 0.29	42.23 ± 0.23	−30.01
Total soluble solids (TSS)	6.193	0.849	13.09 ± 0.11	12.00 ± 0.14	−8.30

# Magnitude of increased (+) or decreased (−), Relative contribution of the divergence characters (Singh, [26]) calculated with distance weighted QMR procedure.

**Table 8:** Traits prioritization for salinity stress tolerance in onions through stepwise regression approach.

Dependent Variable	Step and Variables	R^2^	F-Stat.	Probability
BYP (Bulb yield/plant)	1. BD	0.805	165.014	0.000
	2. BD + PH	0.872	132.617	0.000
	3. BD + PH + APX	0.890	102.647	0.000
	4. BD + PH + APX + gS	0.926	115.676	0.000
	5. BD + PH + APX + gS + POX	0.944	122.048	0.000
	6. BD + PH + APX + gS + POX + CAT	0.961	141.819	0.000
	7. BD + PH + APX + gS + POX + CAT + MDA	0.967	140.697	0.000
	8. BD + PH + APX + gS + POX + CAT + MDA + MSI	0.972	141.141	0.000
	9. BD + PH + APX + gS + POX + CAT + MDA + MSI + Bulb Na^+^/K^+^	0.976	143.261	0.000

BD: Bulb diameter, PH: Plant height, APX: Ascorbate peroxidase, gS: Stomatal conductance, POX: Peroxidase, CAT: Catalase, MDA: Malondialdehyde, MSI: Membrane Stability Index, Bulb: Na^+^/K^+^**.**

## Data Availability

All the relevant data of the study is provided in the manuscript and Supplementary Files.

## Supplementary Materials

The following supporting information can be downloaded at: https://www.mdpi.com/article/10.3390/plants11233325/s1.
