# Supplementary material for: Salt Tolerance Potential in Onion: Confirmation through Physiological and Biochemical Traits
Source: Plants (Basel). 2022 Dec 1;11(23):3325. doi: 10.3390/plants11233325 (PMC9739851; doi:10.3390/plants11233325)
Supplement: Supplementary file 1 [file plants-11-03325-s001.zip › plants-1981001-supplementary.pdf]

# Supplymentary Tables

**Table S1:**Bulb yield and yield related observations of 36 onion accessions under control and salinity stress condition

| Cultivar name* | Control | | | | | Salinity (ECiw7 dSm$^{-1}$) | | | | |
|---|---|---|---|---|---|---|---|---|---|---|
| | Plant height cm) | Number of leaves/plant | Bulb diameter (cm) | Bulb yield/plant (g) | Total soluble solids | Plant height cm) | Number of leaves/plant | Bulb diameter (cm) | Bulb yield/plant (g) | Total soluble solids |
| Pusa Red | 43.17±1.25 | 8.45±0.03 | 5.64±0.09 | 52.29±0.45 | 13.90±0.06 | 41.18±0.28 | 8.10±0.06 | 5.21±0.05 | 36.83±0.07 | 13.20±0.12 |
| Kalyanpur Red Round (KKR) | 42.25±0.42 | 7.90±0.05 | 5.06±0.02 | 40.00±0.14 | 15.77±0.06 | 37.46±0.32 | 7.84±0.09 | 4.21±0.05 | 22.60±0.35 | 14.85±0.04 |
| ALR | 44.88±0.68 | 8.76±0.06 | 4.70±0.06 | 41.53±0.06 | 12.90±0.06 | 41.45±0.40 | 8.56±0.12 | 4.17±0.07 | 23.89±0.06 | 12.20±0.12 |
| B-780 (Baswant780) | 38.95±0.13 | 8.39±0.27 | 5.75±0.03 | 71.05±0.03 | 11.83±0.04 | 35.37±0.25 | 8.02±0.05 | 4.75±0.03 | 46.40±0.23 | 10.67±0.06 |
| Agrifound White | 40.66±0.36 | 8.25±0.03 | 5.10±0.03 | 50.22±0.13 | 13.60±0.17 | 35.49±0.61 | 8.10±0.05 | 4.86±0.03 | 28.18±0.10 | 11.97±0.02 |
| Punjab Naroya | 41.48±0.20 | 7.30±0.12 | 5.23±0.07 | 48.42±0.45 | 13.63±0.06 | 38.73±0.19 | 7.08±0.05 | 4.75±0.03 | 32.78±0.13 | 13.90±0.06 |
| GJWO-1 | 43.82±0.25 | 8.17±0.14 | 5.31±0.02 | 58.70±0.17 | 12.37±0.06 | 42.62±0.47 | 7.96±0.03 | 4.94±0.03 | 49.10±0.06 | 10.37±0.04 |
| RO 59 (Arpita) | 44.85±0.41 | 7.78±0.13 | 5.76±0.02 | 46.42±0.24 | 11.87±0.02 | 42.73±0.53 | 7.60±0.13 | 5.36±0.02 | 32.50±0.29 | 10.60±0.12 |
| Arka Pitamber | 47.33±0.32 | 9.10±0.04 | 4.90±0.05 | 36.89±0.06 | 15.67±0.06 | 43.88±0.42 | 8.76±0.08 | 4.15±0.03 | 26.00±0.14 | 15.27±0.16 |
| NHRDF Red (L-28) | 43.95±0.37 | 9.01±0.06 | 5.80±0.05 | 77.00±0.12 | 12.07±0.04 | 43.48±0.42 | 8.98±0.05 | 5.44±0.06 | 65.35±0.20 | 10.67±0.08 |
| Bhima Super | 42.84±0.47 | 8.44±0.03 | 5.22±0.05 | 56.28±0.16 | 12.30±0.17 | 38.60±0.53 | 7.89±0.08 | 4.84±0.03 | 36.44±0.25 | 12.07±0.01 |
| Bhima Raj | 39.89±0.24 | 8.32±0.05 | 5.29±0.06 | 66.31±0.18 | 12.80±0.12 | 38.83±0.23 | 8.25±0.08 | 4.78±0.03 | 43.37±0.21 | 11.63±0.06 |
| Bhima Red | 38.87±0.39 | 7.90±0.09 | 5.21±0.05 | 51.15±0.09 | 12.43±0.06 | 36.52±0.43 | 7.66±0.12 | 4.73±0.02 | 40.82±0.06 | 12.00±0.12 |
| BhimaShweta | 33.44±0.65 | 8.30±0.06 | 5.33±0.05 | 53.84±0.06 | 11.87±0.08 | 32.50±0.17 | 8.05±0.03 | 5.09±0.05 | 40.53±0.12 | 11.70±0.17 |
| Bhima Shakti | 32.33±0.16 | 8.70±0.14 | 5.44±0.05 | 55.17±0.10 | 12.70±0.06 | 30.45±0.51 | 8.33±0.10 | 4.99±0.08 | 35.88±0.07 | 12.07±0.04 |
| POS38 | 38.75±0.18 | 9.06±0.06 | 5.28±0.08 | 69.67±0.06 | 12.10±0.06 | 37.65±0.45 | 8.91±0.05 | 4.87±0.04 | 55.90±0.06 | 11.50±0.12 |
| Arka Bheem | 45.40±0.40 | 8.21±0.33 | 5.19±0.03 | 54.84±0.09 | 14.37±0.12 | 41.82±0.16 | 7.24±0.14 | 4.72±0.05 | 33.11±0.06 | 11.80±0.12 |
| RO-252 | 43.93±0.53 | 8.35±0.09 | 5.91±0.03 | 56.75±0.14 | 12.37±0.14 | 41.57±0.14 | 8.20±0.07 | 5.31±0.10 | 35.69±0.06 | 11.83±0.04 |
| Bhima Dark Red | 45.26±0.43 | 8.74±0.08 | 5.09±0.05 | 72.10±0.06 | 12.17±0.10 | 42.08±0.20 | 8.29±0.04 | 4.06±0.05 | 26.78±0.05 | 11.10±0.06 |
| NHRDF Red-3 (L-652) | 42.37±0.38 | 8.45±0.09 | 5.26±0.06 | 55.10±0.06 | 12.93±0.04 | 39.82±0.48 | 8.30±0.05 | 4.32±0.07 | 31.05±0.03 | 12.07±0.04 |
| BhimaSafed | 38.60±0.37 | 8.56±0.06 | 5.15±0.04 | 43.10±0.06 | 12.17±0.10 | 34.72±0.51 | 8.22±0.11 | 4.35±0.03 | 27.90±0.06 | 11.43±0.08 |
| JRO 11 (GJRO 11) | 46.55±0.38 | 8.65±0.06 | 5.33±0.05 | 53.25±0.14 | 13.50±0.29 | 43.20±0.52 | 8.40±0.09 | 4.78±0.04 | 35.72±0.03 | 12.57±0.02 |
| Bhima Light Red | 34.98±0.09 | 7.42±0.10 | 5.93±0.04 | 68.61±0.06 | 12.17±0.10 | 33.48±0.27 | 7.21±0.08 | 5.30±0.07 | 46.55±0.06 | 11.77±0.12 |
| HOS 4 (Hisar onion 4) | 41.62±0.23 | 9.06±0.02 | 5.81±0.02 | 56.16±0.09 | 14.03±0.02 | 38.62±0.28 | 8.62±0.07 | 5.33±0.17 | 39.63±0.07 | 13.03±0.02 |
| GJWO 3 | 41.93±0.34 | 7.99±0.03 | 5.38±0.03 | 49.30±0.17 | 12.50±0.06 | 37.65±0.45 | 7.87±0.12 | 4.43±0.08 | 28.28±0.16 | 11.00±0.09 |
| NHRDF Red-4 (L-744) | 45.43±0.33 | 8.85±0.03 | 5.92±0.06 | 74.38±0.69 | 12.17±0.10 | 44.03±0.36 | 8.71±0.06 | 5.29±0.05 | 60.40±0.23 | 11.63±0.12 |
| Pusa Shobha | 41.77±0.25 | 7.40±0.12 | 5.37±0.04 | 64.38±0.47 | 16.80±0.03 | 39.90±0.18 | 7.25±0.06 | 5.35±0.05 | 44.12±0.07 | 13.80±0.06 |
| Pusa Sona | 39.48±0.33 | 7.66±0.05 | 5.68±0.09 | 60.20±0.53 | 12.90±0.06 | 37.90±0.34 | 7.60±0.03 | 4.72±0.05 | 37.06±0.03 | 12.17±0.10 |

| | | | | | | | | | | |
|---|---|---|---|---|---|---|---|---|---|---|
| POS35 | 43.22±0.18 | 8.30±0.02 | 6.18±0.02 | 77.48±0.34 | 12.43±0.02 | 42.20±0.25 | 8.27±0.14 | 5.62±0.07 | 65.95±0.03 | 12.00±0.03 |
| POS36 | 43.25±0.45 | 8.35±0.06 | 5.87±0.08 | 66.95±0.12 | 13.37±0.06 | 42.26±0.32 | 8.15±0.08 | 5.02±0.05 | 55.44±0.25 | 12.30±0.17 |
| POS39 | 41.30±0.26 | 8.79±0.05 | 5.81±0.05 | 52.65±0.20 | 15.20±0.12 | 38.33±0.16 | 7.90±0.06 | 4.58±0.03 | 41.83±0.07 | 13.37±0.05 |
| POS37 | 43.20±0.23 | 8.70±0.09 | 5.69±0.30 | 70.65±0.09 | 12.77±0.06 | 42.55±0.26 | 8.48±0.17 | 5.54±0.04 | 56.89±0.06 | 12.07±0.04 |
| Sukhsagar | 40.73±0.44 | 8.12±0.05 | 5.05±0.03 | 51.26±0.15 | 14.50±0.06 | 35.47±0.19 | 7.60±0.12 | 4.04±0.02 | 31.67±0.19 | 11.60±0.12 |
| PRO 6 | 39.60±0.41 | 7.66±0.09 | 5.67±0.08 | 59.79±0.12 | 15.53±0.06 | 38.13±0.20 | 7.60±0.07 | 4.90±0.07 | 39.10±0.06 | 12.57±0.02 |
| JNDWO-85 | 43.27±0.26 | 8.44±0.04 | 5.02±0.04 | 53.10±0.06 | 12.33±0.19 | 40.00±0.60 | 8.36±0.21 | 4.56±0.06 | 38.95±0.03 | 11.17±0.10 |
| NHRDF (L-920) | 46.42±0.32 | 8.08±0.05 | 4.72±0.03 | 41.56±0.12 | 16.10±0.03 | 43.67±0.52 | 8.06±0.03 | 3.50±0.03 | 21.36±0.21 | 14.30±0.17 |

**Table S2**.$Na^+/K^+$ ratios of selected onion accessions under control and salinity stress

| Cultivars name | Root $Na^+/K^+$ | | Shoot $Na^+/K^+$ | | Bulb $Na^+/K^+$ | |
|---|---|---|---|---|---|---|
| | **Control** | **Salinity** | **Control** | **Salinity** | **Control** | **Salinity** |
| POS35 | 1.29±0.07$^{f}$ | 3.25±0.25$^{cde}$ | 1.13±0.03$^{de}$ | 2.35±0.03$^{g}$ | 0.21±0.02$^{cd}$ | 0.30±0.02$^{de}$ |
| L-28 | 1.57±0.06$^{de}$ | 3.29±0.09$^{bcd}$ | 0.89±0.04$^{f}$ | 1.65±0.04$^{i}$ | 0.25±0.01$^{ab}$ | 0.31±0.02$^{de}$ |
| GJWO 1 | 1.15±0.07$^{fg}$ | 3.10±0.08$^{e}$ | 1.05±0.03$^{e}$ | 2.42±0.10$^{g}$ | 0.26±0.03$^{a}$ | 0.41±0.02$^{a}$ |
| POS36 | 1.55±0.05$^{e}$ | 3.10±0.04$^{e}$ | 1.67±0.05$^{a}$ | 2.36±0.04$^{g}$ | 0.23±0.02$^{abc}$ | 0.26±0.01$^{f}$ |
| POS37 | 1.30±0.04$^{f}$ | 3.31±0.07$^{bcd}$ | 1.06±0.06$^{e}$ | 1.44±0.10$^{j}$ | 0.23±0.01$^{abc}$ | 0.36±0.03$^{b}$ |
| L-744 | 1.28±0.05$^{f}$ | 3.20±0.05$^{cde}$ | 0.95±0.05$^{f}$ | 1.92±0.08$^{h}$ | 0.25±0.02$^{ab}$ | 0.29±0.02$^{def}$ |
| POS38 | 2.16±0.11$^{b}$ | 3.32±0.10$^{bcd}$ | 1.46±0.10$^{b}$ | 2.64±0.04$^{f}$ | 0.23±0.01$^{abc}$ | 0.28±0.02$^{ef}$ |
| ALR | 2.07±0.07$^{b}$ | 3.46±0.16$^{b}$ | 1.37±0.07$^{c}$ | 3.88±0.08$^{a}$ | 0.26±0.01$^{a}$ | 0.31±0.01$^{de}$ |
| GJWO 3 | 1.71±0.10$^{cde}$ | 3.38±0.08b$^{c}$ | 1.45±0.03b$^{c}$ | 3.78±0.06$^{b}$ | 0.25±0.01$^{ab}$ | 0.32±0.03$^{cd}$ |
| KRR | 2.48±0.18$^{a}$ | 3.79±0.11$^{a}$ | 1.13±0.05d$^{e}$ | 3.79±0.04$^{b}$ | 0.22±0.02$^{bcd}$ | 0.38±0.04$^{ab}$ |
| L-652 | 1.76±0.04$^{c}$ | 3.76±0.11$^{a}$ | 1.59±0.03$^{a}$ | 3.63±0.15$^{c}$ | 0.24±0.01$^{abc}$ | 0.36±0.03$^{b}$ |

| | | | | | | |
|---|---|---|---|---|---|---|
| Agrifound White | 1.73±0.05$^{cd}$ | 3.66±0.06$^{a}$ | 1.13±0.05d$^{e}$ | 3.66±0.05$^{c}$ | 0.19±0.02$^{d}$ | 0.30±0.01$^{de}$ |
| L-920 | 1.05±0.05$^{g}$ | 3.29±0.09$^{bcd}$ | 0.71±0.02$^{g}$ | 2.76±0.06$^{e}$ | 0.24±0.01$^{abc}$ | 0.35±0.03$^{bc}$ |
| Bhima Dark Red | 2.13±0.03$^{b}$ | 3.14±0.14$^{de}$ | 1.21±0.03$^{d}$ | 3.47±0.07$^{d}$ | 0.19±0.01$^{d}$ | 0.37±0.01$^{b}$ |

Numerical Values are represented as Mean ± SD, means comparison was performed through, Duncan's Multiple Range Test (DMRT), Values with the same letter as super script are not significantly different.

**Table S3.** Regression coefficient, standard error, and significance of the prioritized traits entered into the predicted response for salinity stress tolerance

| **Variables in equation** | **Regression coefficients (βs)** | **Standard Error(SE)** | **t -Value** | **Pr(>\|t\|)** | **F-to-Remove** |
|---|---|---|---|---|---|
| Constant | -409.75 | | | | |
| BD | 21.38 | 1.91 | 11.18 | 0.000 | 125.06 |
| PH | 2.28 | 0.20 | 11.10 | 0.000 | 123.25 |
| APX | 0.52 | 0.06 | 8.24 | 0.000 | 67.85 |
| gS | -137.77 | 39.75 | -3.47 | 0.002 | 12.01 |
| POX | 1.21 | 0.23 | 5.21 | 0.000 | 27.17 |
| CAT | -1.47 | 0.52 | -2.85 | 0.008 | 8.14 |
| MDA | 3.21 | 0.72 | 4.45 | 0.000 | 19.82 |
| MSI | 1.44 | 0.42 | 3.44 | 0.001 | 11.81 |
| Bulb $Na^+/K^+$ | 33.57 | 14.29 | 2.35 | 0.025 | 5.52 |

**Predicted bulb yield = −409.75+ (21.38 × BD) + (2.28 × PH) + (0.52× APX) + (−137.77× gS) +(1.21 × POX) + (-1.47 × CAT) + (3.21 × MDA) + (1.44 × MSI) + (Bulb $Na^+$ /$K^+$)**

#BD-Bulb diameter, PH-Plant height, APX-Ascorbate peroxidase, gS-Stomatal conductance, POX-Peroxidase, CAT-Catalase, MDA-Malondialdehyde, MSI-Membrane Stability Index, Bulb $Na^+$ /$K^+$
